# *DkNAC7*, a novel high-CO_2_/hypoxia-induced NAC transcription factor, regulates persimmon fruit de-astringency

**DOI:** 10.1371/journal.pone.0194326

**Published:** 2018-03-14

**Authors:** Rong Jin, Qing-gang Zhu, Xin-yue Shen, Miao-miao Wang, Wajeeha Jamil, Donald Grierson, Xue-ren Yin, Kun-song Chen

**Affiliations:** 1 Zhejiang Provincial Key Laboratory of Horticultural Plant Integrative Biology, Zhejiang University, Zijingang Campus, Hangzhou, PR China; 2 Agricultural Experiment Station, Zhejiang University, Zijingang Campus, Hangzhou, PR China; 3 Plant & Crop Sciences Division, School of Biosciences, University of Nottingham, Sutton Bonington Campus, Loughborough, United Kingdom; 4 The State Agriculture Ministry Laboratory of Horticultural Plant Growth, Development and Quality Improvement, Zhejiang University, Zijingang Campus, Hangzhou, PR China; Institute of Genetics and Developmental Biology Chinese Academy of Sciences, CHINA

## Abstract

Artificial high-CO_2_ atmosphere (AHCA, 95% CO_2_ and 1% O_2_) has been widely applied as a postharvest de-astringency treatment for persimmon fruit. AHCA increases expression of transcription factors, including ethylene response factors (*DkERF*), that target de-astringency genes. Here, the promoter of *DkERF9*, a previously characterized AHCA-inducible and de-astringency regulator, was utilized to screen a cDNA library by yeast one hybrid assay. A novel NAC transcription factor, named *DkNAC7*, was identified. Dual-luciferase assay indicated that *DkNAC7* could not only trans-activate the promoter of *DkERF9*, but also activated the previously identified deastringency-related gene *DkPDC2*. Real-time PCR analysis showed that *DkNAC7* was up-regulated by AHCA treatment, in concert with the removal of astringency from persimmon fruit and subcellular localization showed *DkNAC7* was located in the nucleus. Thus, these results indicate that *DkNAC7* is a putative transcriptional activator involved in regulating persimmon fruit deastringency by trans-activition on both *DkERF9* and *DkPDC2*, which encodes pyruvate decarboxylase.

## Introduction

Persimmon (*Diospyros kaki* L.) is a worldwide crop, which originated in Southeast Asia. Persimmon fruit can be divided into astringent and non-astringent types, but most native cultivars in China are of the astringent types [1]. These astringent persimmon fruit have the unique feature of accumulating abundant amounts of condensed tannins (CT) [2]. Astringent persimmon accumulates abundant CTs in fruit even at maturity and soluble CTs (SCT) cause astringency [1,3], which severely affects the persimmon industry and consumer acceptance.

A range of artificial technologies have been developed to remove astringency, including high-CO_2_, ethylene and ethanol [4–8]. Among these, high CO_2_ (usually > 90%) is the most widely used treatment, in which the O_2_ level is reduced to 1%. In plants, hypoxia usually causes the accumulation of products from anaerobic metabolism [9], and these products (especially acetaldehyde) effectively reduce the SCTs and accelerate deastringency in persimmon fruit [5,7,10]. The activities of alcohol dehydrogenase (ADH, EC 1.1.1.1) and pyruvate decarboxylase (PDC, EC 4.1.1.1) and also their encoding genes (*DkADH1*, *DkPDC1*, *DkPDC2* and *DkPDC3*) have been shown to increase in amount during deastringency [7,11]. Transient over-expression of *DkPDC2* led to a lower level of SCTs in persimmon leaves [7], suggesting that *DkPDC2* is a key gene for the de-astringency program of persimmon fruit. These results confirmed that CO_2_ driven astringency removal involves hypoxia-triggered acetaldehyde metabolism.

In the model plant *Arabidopsis*, a few ethylene response factors (*ERFs*) have been reported to be involved in the hypoxia response, including *HRE1*, *HRE2*, *RAP2*.*2* and *RAP2*.*12*. These *ERF* genes could transcriptionally regulate *ADH* and *PDC*, and result in hypoxia tolerance [12–15]. As stated above, persimmon fruit deastringency by high CO_2_ treatment is considered to operate mainly through the hypoxia fermentation pathway. In persimmon, four *DkERF* were previously reported to be involved in persimmon fruit deastringency, including *DkERF9/10/19/22* [7,8]. Of these, *DkERF9* was characterized as an activator of the promoter of *DkPDC2*, a key gene for deastringency [7]. Due to the low oxygen in high CO_2_ treatment, these *DkERFs* were previously termed as hypoxia responsive [8]. But, high CO_2_ treatment is an atypical anoxia environment, with effects of both high CO_2_ and low O_2_, thus it could be induced either a high-CO_2_ or hypoxia response.

Apart from *ERFs*, some other transcription factors were reported as high-CO_2_/hypoxia responsive in persimmon fruit, such as *DkMYB6* [16] and *DkTGA1* [17]. *NAC* genes are the main transcription factors reported to be involved in the plant hypoxia response. In *Arabidopsis*, more than 100 *NAC* genes have been characterized [18] that share highly conserved consensus in the N-terminal region of a Petunia gene (NAM), *Arabidopsis* ATAF1/2 and CUC2 proteins [19]. Among these genes, hypoxia-responsive *NAC* genes have rarely been reported, and the results from studies on *ANAC102* also indicate that additional *NAC* genes might exist for the hypoxia response, as *ANAC102* knockout lines did not show altered *ADH* gene transcription in *Arabidopsis* [9]. In persimmon, six *NAC* genes have been characterized, among which *DkNAC1/3/5/6* were high-CO_2_/hypoxia responsive, however their regulatory roles in persimmon deastringency remain unclear [20]. Thus, the potential role of *NAC* genes in regulating persimmon deastringency still lacks experimental evidence.

Here, a novel NAC transcription factor (*DkNAC7*) was obtained as a result of yeast one hybrid screening by using the promoter of *DkERF9* as bait and the regulatory role of *DkNAC7* in persimmon de-astringency was investigated using yeast one-hybrid assay, dual-luciferase, real-time PCR and subcellular localization.

## Materials and methods

### Plant materials and treatment

‘Mopanshi’ (astringent cultivar) persimmon (*D*. *kaki*) fruit were obtained from a commercial orchard at Fangshan (Beijing, China) in 2012. Fruit without disease or mechanical wounding were selected and treated with artificial high-CO_2_ atmosphere (AHCA, 95% CO_2_ and 1% O_2_) or air in air-tight containers for 1 d. The physiological data and sampling information were described in Wang et al. [21].

### RNA extraction and cDNA synthesis

Total RNAs were extracted from frozen fruit flesh (2.0 g) and the cDNA synthesis carried out according to the method used previously [6].

### Gene isolation and sequence analysis

A NAC transcription factor was obtained based on the Matchmaker Gold Yeast One-hybrid Library Screening System (Clontech, USA), using the promoter of deastringency-related *DkERF9* [7,16] as the bait DNA sequence. The full-length *NAC* gene was isolated with a SMART RACE cDNA Amplification Kit (Clontech). The sequences of primers are described in Table 1. For phylogenetic analysis, the NAC genes in persimmon and methods were as described in Min *et al* [20]

**Table 1:** Sequences of the primers used for RACE, full-length amplification, real time PCR and vector construction.

Gene	Methods used	Primers (5’-3’)
DkNAC7	3’RACE (Primary PCR)	CAAGCCCTTCCGATTCGATGCCAT
DkNAC7	3’RACE (Secondary PCR)	GGAAGACAACAGGAAAGGACAGGCC
DkNAC7	5’RACE (Primary PCR)	CTCGTCATCCTCCCATTCCTCCTCAAC
DkNAC7	5’RACE (Secondary PCR)	CTCGTGCATCACCCAGTTGGTCCTCT
DkNAC7	Full-length clone (FP)	CATCGGCGGTGACCAAAACGG
DkNAC7	Full-length clone (RP)	CACAAAGTCCCTAGATCTCAGA
DkNAC7	Y1H constructs (FP)	CGCGAATTCATGGGCCTCGATCCATCGTC
DkNAC7	Y1H constructs (RP)	GATGGATCCCTACCTCGATGCATTTCCCG
DkNAC7	SK vector construction (FP)	CGCGCGGCCGCATGGGCCTCGATCCATCGTC
DkNAC7	SK vector construction (RP)	GATGGATCCCTACCTCGATGCATTTCCCG
DkNAC7	Q-PCR (FP)	TGAGTTTCAAAATTGGGAGT
DkNAC7	Q-PCR (RP)	CCCTAGATCTCAGATGGTGA
DkNAC7	GFP vector construction (FP)	CGCGGTACCATGGGCCTCGATCCATCGTC
DkNAC7	GFP vector construction (RP)	CATGTCGACCCTCGATGCATTTCCCG
DkERF9	pAbAi vector construction (FP)	CGCGAGCTCAAATAATTTAATTAAAGATA
DkERF9	pAbAi vector construction (RP)	CGCGTCGACATACACAGGAAAACAGGATT

Note: underlined sequences show cutting sites for restriction enzymes

### Yeast one-hybrid assay (Y1H)

According to library screening results, the protein-DNA interaction was verified with DkNAC7-AD and *DkERF9* promoter, individually. Meanwhile, interaction between DkNAC7 and *DkPDC2* promoter was also investigated by Y1H. The promoter of *DkERF9* was constructed into pAbAi vector (primers are listed in Table 1). The *DkPDC2*-pAbAi was constructed by Min *et al*. [8]. The full-length sequence of transcription factor *DkNAC7* was subcloned into pGADT7 AD vector (AD) (primers are listed in Table 1).

Auto-activation and the interaction analysis were conducted according to the manufacturer’s protocol.

### Dual luciferase assay

Dual-luciferase assay was used as a rapid and efficient method to detect *in vivo* trans-activation or trans-repression effects of transcription factors [22]. Full-length *DkNAC7* was inserted into pGreen II 0029 62-SK vector (SK), using the primers listed in Table 1. The dual luciferase assay was carried out with *Nicotiana benthamiana* leaves, using the protocol described by Min *et al*. [7]. Three independent experiments (with minimum five replicates) were performed to verify the results.

### Real-time PCR analysis

For real-time PCR, gene specific oligonucleotide primers were designed and are shown in Table 1. The quality and specificity of primers were checked by melting curve and PCR products resequencing. The *DkACT* was chosen as a housekeeping gene to monitor the abundance of mRNA [7].

Real-time PCR reactions were carried out on a CFX96 instrument (Bio-Rad). The PCR protocols were according to our previous reports, using Ssofast EvaGreen Supermix Kit (Bio-Rad) [6]. The relative expression of this *NAC* gene was calibrated with values for day 0 fruit set as 1.

### Subcellular localization analysis

35S-*DkNAC7*-GFP was transiently expressed in tobacco leaves by *Agrobacterium*-mediated infiltration (GV3101) according to previous reports [23,24]. The green fluorescent protein (GFP) fluorescence in tobacco leaves was imaged 2 d after infiltration using a Zeiss LSM710NLO confocal laser scanning microscope. Primers used for GFP construction are described in Table 1.

### Statistical analysis

Least Significant Difference (LSD) test was used to compare the statistical significance differences among treatments by using DPS 7.05 or Student’s *t*-test. The figures were drawn with Origin 8.0.

## Results and discussion

### Y1H based library screening discovered a novel *NAC* gene, which targeted the *DkERF9* promoter

**Fig 1:**
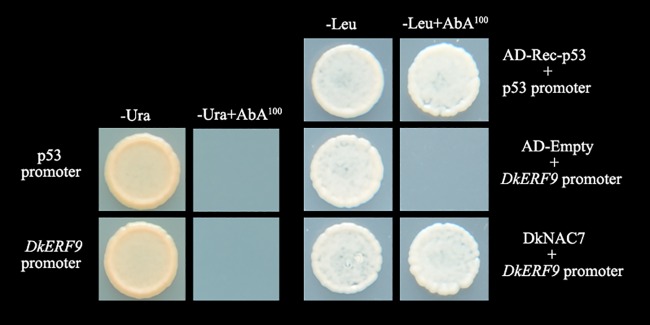
Protein-DNA interaction between DkNAC7 and the promoter of *DkERF9* using yeast one hybrid analysis. Interaction was confirmed on SD medium lacking Leu in the presence of aureobasidin A (-Leu+AbA^100^). AD-Rec-p53 and p53-AbAi were used as a positive control; AD-empty and pDkERF9-AbAi were used as a negative control.

In our previous reports, *DkERF9* transcription factor was shown to be involved in persimmon de-astringency via regulation of the *DkPDC2* promoter [7]. In order to obtain further information about the transcriptional regulatory mechanism controlling persimmon fruit deastringency, Y1H based screening was employed to screening the potential interacting transcription factors, using the *DkERF9* promoter as bait. A total of 150 PCR products were obtained, among which only one NAC transcription factor gene was characterized. Individual verifications with Y1H indicated that the NAC transcription factor could bind to *DkERF9* promoter (Fig 1). As six *DkNAC* genes were reported previously in persimmon fruit [20], this NAC transcription factor was named as *DkNAC7* (GenBank no.MG792350) (Fig 1).

**Fig 2:**
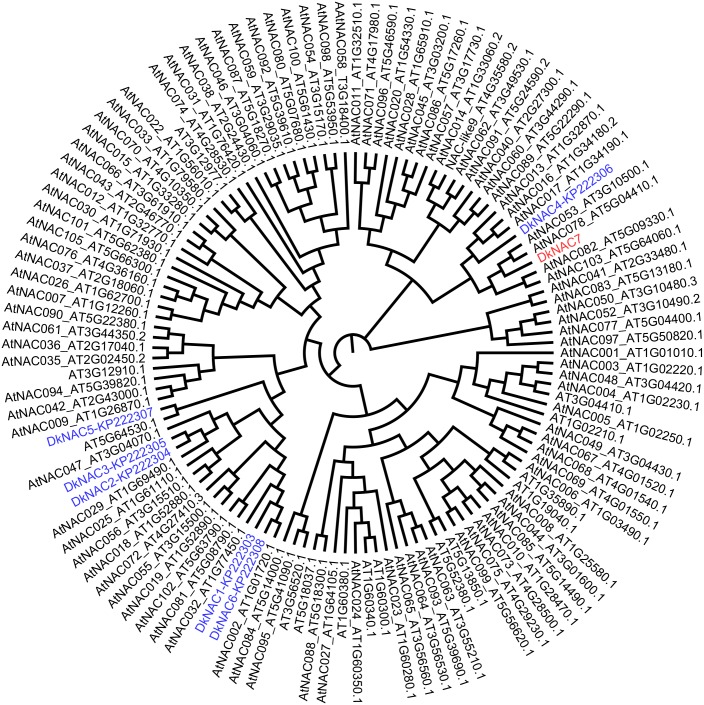
Phylogenetic analysis of DkNAC7 and NAC members from persimmon and *Arabidopsis*. Persimmon *DkNAC* is highlighted in red (*DkNAC7*, newly isolated) and blue (previously reported). The amino acid sequences of NAC transcription factors were obtained from the *Arabidopsis* Information Resource or National Center for Biotechnology Information, and their accession numbers are included in the diagram. The phylogenetic tree was constructed with Figtree (v 1.3.1).

Phylogenetic tree analysis indicated that *DkNAC7* was close to *DkNAC4*, but not the other five previously reported *DkNAC* genes (Fig 2) [20]. Compared to *Arabidopsis* NAC transcription factors, *DkNAC7* was close to *AtNAC078*, which was reported to regulate flavonoid biosynthesis under high light in *Arabidopsis* [25], while it was not clustered with *ANAC102*, which was shown to be induced by low oxygen (0.1%) in *Arabidopsis* [9].

### *In vivo* regulatory roles of DkNAC7 on deastringency related genes (*DkERF*, *DkADH1* and *DkPDC2*)

**Fig 3:**
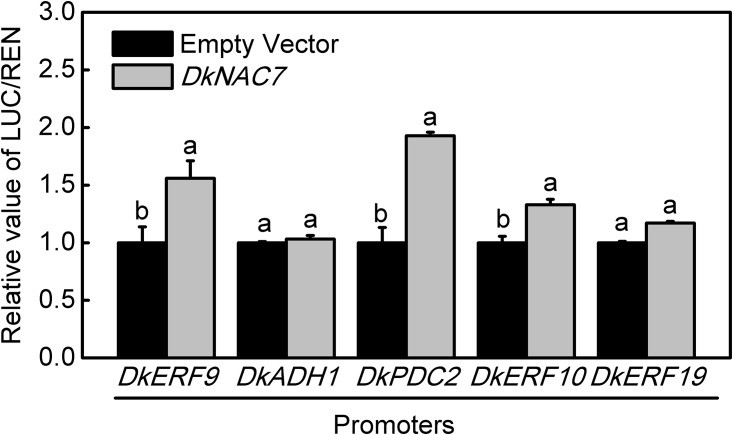
Regulatory effects of DkNAC7 on the promoters of deastringency-related genes (*DkERF9/10/19*, *DkADH1*, *DkPDC2*) using the dual-luciferase assay. The ratio of LUC/REN of the empty vector (SK) plus promoter was used as calibrator (set as 1). Error bars indicate SEs from five replicates. Different letters above the columns indicate significant differences (*P<0*.*05*).

Further investigations on the possible transcriptional regulatory linkage between *DkNAC7* and deastringency related genes were carried out. Three previously studied *DkERF* genes (*DkERF9*/*10/19*) and two structural genes (*DkADH1* and *DkPDC2*) were selected for test. Dual luciferase assay indicated that *DkNAC7* could significantly trans-activate the promoters of *DkERF9* and *DkPDC2* with 1.55 and 1.92-fold enhancement, respectively (Fig 3). The effect of DkNAC7 on the *DkERF10* promoter also reached statistical significance, but the response was very limited (about 1.32-fold) and the *DkNAC7* gene had no significant effects on the promoters of *DkADH1* and *DkERF19* (Fig 3).

In persimmon, twenty-two ethylene response factors (*DkERFs*) were differently expressed in response to high CO_2_ treatment. Of these 22 genes, only four *ERFs* (*DkERF9/10/19/22*) were fund to be involved in persimmon de-astringency [7,8], via the interaction with promoters of de-astringent related target genes (e.g. *DkADH1*, *DkPDC2* and *DkPDC3*). Furthermore, a MYB transcription factor (*DkMYB6*) and a bZIP transcription factor (*DkTGA1*) were also characterized as regulators of persimmon fruit astringency removal, respectively [16,17]. Thus, these finding with *DkNAC7* unveiled a new transcription factor that participates in regulation of persimmon fruit deastringency. Furthermore, *DkNAC7* is not closely related to the low oxygen-induced *ANAC102* gene [9] from phylogenetic result (Fig 2) which suggests that more than one various type NAC transcription factor may contribute to the hypoxia response.

### Cascade regulations of *DkNAC7*-*DkERF9*-*DkPDC2*

Comparing the effects *DkNAC7* and the previously characterized TFs on the *DkPDC2* promoter, *DkNAC7* was shown to have only a relatively limited action, which was only slightly stronger than *DkTGA1* [17]. Y1H analyses indicated that *DkNAC7* cannot bind to and is therefore an indirect regulator for hypoxia responsive *DkPDC2* (S1 Fig). As the present results indicated that interaction between DkNAC7 and *DkERF9* promoter (Fig 1) and our previous study indicated that DkERF9 could physically bind to *DkPDC2* promoter [17]. Thus, it could be proposed that a regulatory cascade involving *DkNAC7-DkERF9-DkPDC2* contributes to persimmon fruit deastringency. The regulatory roles of NAC transcription factors in hierarchical interactions with *ERFs* have also been reported in other fruits, for instance *MdNAC029/MdNAP*, an apple NAC gene, was reported to directly repressed the expression of two *ERF* genes (*MdCBF1* and *MdCBF4*) by binding to their promoters, thus negatively regulating cold tolerance via the *CBF*-dependent pathway [26]. These finding from persimmon not only partial explain the transcriptional regulations during deastringency, but also provided a new example of NAC-ERF regulation. Moreover, since the NAC-ERF cascade contributes to persimmon deastringency (high-CO_2_/hypoxia response) and apple cold tolerance, this raises the question whether other NAC-ERF may be involved in abiotic stress responses.

### Expression and subcellular localization analyses for *DkNAC7*

**Fig 4:**
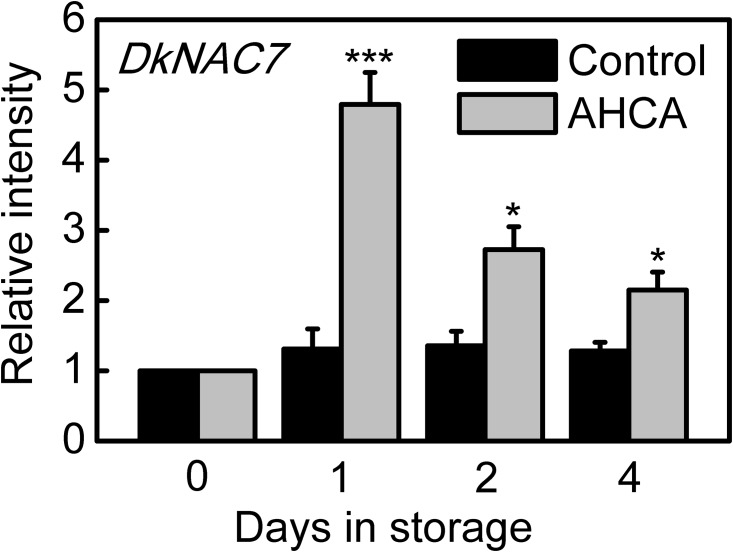
Expression of *DkNAC7* in response to AHCA treatment (95% CO_2_, 1% O_2_, 1 day). Relative mRNA abundance was evaluated by real-time PCR. Day 0 fruit values were set as 1. Error bars represent ± SE from three replicates (*p < 0.05; ***p < 0.001).

**Fig 5:**
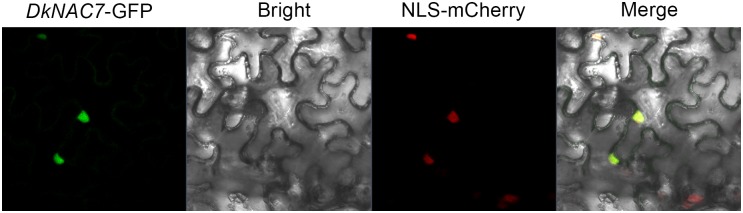
Subcellular localization of *DkNAC7*-GFP in tobacco leaves transformed by agroinfiltration. GFP fluorescence of DkNAC7 is indicated. Bars = 25 μm.

The above-mentioned regulatory effects of *DkNAC7* on deastringency related genes encouraged us to study the response of *DkNAC7* to deastringency treatment. From previous results, AHCA treatment (also called CO_2_ treatment or high CO_2_ treatment: 95% CO_2_ and 1% O_2_) was very effective in removing astringency in various persimmon [5,7,21]. Therefore, using previously described materials [21], the expression of *DkNAC7* was analyzed. The *DkNAC7* gene exhibited a sharp increase in expression in response to AHCA treatment, with the highest level at 1 d (Fig 4). After removal of CO_2_ treatment, transcripts of *DkNAC7* decreased concomitantly, but remained statistically significantly higher than in control fruits. Such expression was similar to most of the previously identified deastringency related transcription factors. Furthermore, subcellular localization analysis of *DkNAC7* in tobacco leaves using GFP tagging, showed strong signals in the nucleus (Fig 5).

Taken all the results of Y1H, dual-luciferase assay, expression and subcellular localization together, we propose *DkNAC7* as a novel regulator of persimmon fruit deastringency, acting via direct regulation of the *DkERF9*. Again, *DkNAC7* was not closest homolog to the low oxygen-induced *ANAC102* gene [9], indicating either potential differences between species or organs, or the complexity of NAC-regulatory roles. Another possible explanation would be the differences between experimental treatments, as in model plant or crops, anoxia treatments were generally low O_2_, but AHCA treatment in persimmon involves high CO_2_ and low O_2_. Thus, the deastringency related *DkNAC7*, as well as the previously characterized transcription factors, could be termed as high-CO_2_/hypoxia responsive.

## Supporting information

S1 Fig Yeast one-hybrid analysis of DkNAC7 binding to promoter of *DkPDC2*. (TIF)

## Abbreviations

AbA: aureobasidin A
AD: pGADT7
ADH: alcohol dehydrogenase
cv: Cultivar
ERF: ethylene response factor
PDC: pyruvate decarboxylase
SCT: soluble condensed tannins
