# Supplementary figures and images for: DkNAC7, a novel high-CO2/hypoxia-induced NAC transcription factor, regulates persimmon fruit de-astringency
Source: PLoS One. 2018 Mar 14;13(3):e0194326. doi: 10.1371/journal.pone.0194326 (PMC5851633; doi:10.1371/journal.pone.0194326)

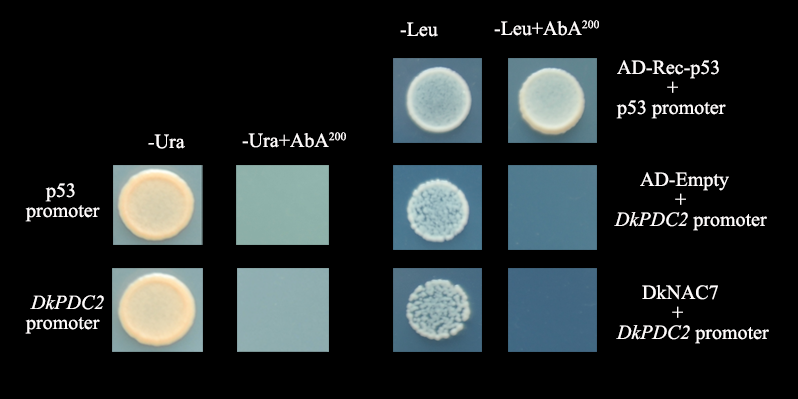

Supplement: S1 Fig — (TIF) [file pone.0194326.s001.tif]
